# Late-stage leukemias in aging populations: a public health call for integrated and personalized care models

**DOI:** 10.1097/MS9.0000000000004412

**Published:** 2025-11-25

**Authors:** Emmanuel Ifeanyi Obeagu

**Affiliations:** Department of Biomedical and Laboratory Science, Africa University, Mutare, Zimbabwe

**Keywords:** aging population, integrated care, late-stage leukemia, personalized medicine, public health

## Abstract

Late-stage leukemias in older adults present a complex interplay of adverse disease biology, age-related vulnerabilities, and health care system challenges. Aging populations face unique clinical and public health burdens, including frailty, comorbidities, and limited access to specialized care, which complicate standard leukemia management. This narrative review synthesizes current evidence on late-stage acute and chronic leukemias in aging populations, highlighting age-specific biological features, therapeutic options, supportive-care considerations, and integrated care strategies. A structured literature search of PubMed, Scopus, Web of Science, and Google Scholar (2000–2025) was conducted using key terms including “*late-stage leukemia*,” “*elderly AML/ALL/CLL/CML*,” “*geriatric assessment*,” and “*integrated care*.” Eligible publications included clinical trials, observational studies, reviews, and policy documents focusing on adults aged ≥60 years. Evidence was synthesized narratively to provide an integrative perspective. Late-stage leukemias in older adults are characterized by higher prevalence of adverse genetic profiles, increased treatment-related toxicity, and reduced tolerance to intensive regimens. Age-adapted therapeutic strategies – including hypomethylating agents, targeted therapies, immunotherapies, and tyrosine kinase inhibitors – can improve outcomes when combined with comprehensive geriatric assessment and supportive care. Integrated care models incorporating multidisciplinary teams, early palliative interventions, and patient-centered decision-making optimize functional status, quality of life, and health care utilization. Effective management of late-stage leukemia in older adults requires personalized, multidisciplinary approaches informed by disease biology, geriatric assessment, and patient priorities.

## Introduction

The global population is aging rapidly, with a concomitant rise in cancer incidence, including hematological malignancies such as leukemia. Late-stage leukemias in elderly patients pose unique challenges distinct from those encountered in younger cohorts. This demographic shift necessitates a reevaluation of clinical strategies, health system organization, and public health policies to adequately address the growing burden^[1,2]^. Leukemia in older adults often presents at advanced stages due to delayed diagnosis, nonspecific symptoms, and age-related physiological changes that mask early disease. Late-stage presentation is associated with more aggressive disease biology, increased resistance to therapy, and higher rates of treatment-related complications. The coexistence of multiple comorbidities, polypharmacy, and diminished functional reserve further complicates management and negatively impacts prognosis^[3,4]^. Current treatment paradigms predominantly designed for younger, fitter patients are often unsuitable for aging populations. Conventional intensive chemotherapy and hematopoietic stem cell transplantation may be contraindicated or poorly tolerated, necessitating alternative, less toxic regimens. However, therapeutic nihilism persists due to the lack of robust clinical trial data specifically targeting elderly patients with late-stage leukemia^[5]^.

**HIGHLIGHTS**

- Late-stage leukemias in the elderly demand age-tailored, integrative health care approaches.
- Geriatric assessments improve treatment decisions and reduce toxicity.
- Personalized therapies enhance tolerability and outcomes.
- Multidisciplinary teams ensure holistic care delivery.
- Early palliative care integration improves quality of life and survival.

Moreover, health care delivery models are frequently fragmented, with inadequate integration between oncology, geriatrics, palliative care, and social support services. This siloed approach undermines holistic patient management, leading to unmet needs and suboptimal quality of life. Personalized medicine, incorporating molecular profiling and geriatric assessment, offers promising pathways to tailor treatment and supportive care according to individual patient characteristics^[6,7]^. Public health systems must adapt to these evolving demands by implementing integrated care models that foster multidisciplinary collaboration and patient-centered approaches. Policies must ensure equitable access to diagnostics, therapies, and supportive resources, particularly for vulnerable elderly populations who often face socioeconomic and geographic barriers. Investment in workforce training, infrastructure, and research focused on aging leukemia patients is critical for sustainable improvements^[8]^. This review explores the clinical and public health dimensions of late-stage leukemias in aging populations, emphasizing the need for integrated, personalized care models. It highlights current challenges, innovations, and strategic recommendations aimed at enhancing care delivery and outcomes for this growing patient cohort.

Leukemias collectively account for approximately **3% of all new cancer diagnoses worldwide**, with an estimated **475 000 new cases and 311 000 deaths annually** according to the 2022 GLOBOCAN database. The incidence of **acute myeloid leukemia (AML)** rises sharply with age, with median diagnosis occurring at **68–72 years** in high-income countries, while **chronic lymphocytic leukemia (CLL)** shows the most pronounced age gradient, with over **70% of cases diagnosed after 65 years**. Regional disparities reflect both demographic patterns and health care capacity: age-standardized incidence rates for AML and CLL are highest in **North America and Western Europe**, where life expectancy is longer and diagnostic infrastructure is robust, whereas **East and South Asia** report lower crude rates but face rapidly rising incidence as population’s age. Low- and middle-income countries (LMICs), particularly in **sub-Saharan Africa and parts of South Asia**, experience underdiagnosis and delayed presentation, often with more advanced or “late-stage” disease due to limited access to molecular testing and specialized care. Mortality-to-incidence ratios remain disproportionately high in LMICs, underscoring the urgent need for region-specific strategies that strengthen diagnostic capacity, equitable access to targeted therapies, and age-adapted supportive care to meet the growing global burden of late-stage leukemias in aging populations.^7−8^

## Aim

This review aims to explore the clinical, diagnostic, and therapeutic complexities associated with late-stage leukemias in aging populations, highlighting the urgent need for integrated and personalized care models. It discusses current challenges in managing leukemia among elderly patients, examines public health implications, and proposes strategic frameworks to improve outcomes through tailored interventions and multidisciplinary collaboration.

## Methods

This narrative review was conducted using a structured, transparent approach to identify and synthesize current evidence on late-stage leukemias in aging populations. Literature searches were performed across PubMed, Scopus, Web of Science, and Google Scholar from January 2000 to September 2025. Search terms included combinations of “late-stage leukemia,” “elderly,” “aging population,” “geriatric oncology,” “integrated care,” “personalized medicine,” and “public health models.” Additional articles were identified through manual screening of reference lists and relevant review papers.

Peer-reviewed original studies, systematic reviews, meta-analyses, clinical guidelines, and policy documents were included if they addressed epidemiology, biological mechanisms, diagnostic challenges, therapeutic approaches, or health care delivery models relevant to older adults with advanced leukemia. Non-English articles, conference abstracts without full text, commentaries, and publications unrelated to late-stage disease or aging populations were excluded.

All eligible articles were screened by the authors for relevance and methodological adequacy. Extracted information was organized thematically to guide narrative synthesis, focusing on epidemiologic trends, biological characteristics in aging individuals, diagnostic barriers, therapeutic complexities, integrated care frameworks, and innovations in personalized medicine. The goal was to provide a comprehensive and coherent overview rather than a quantitative meta-analysis.

## Definition of “late-stage leukemia”

For the purposes of this manuscript, **“late-stage leukemia”** denotes leukemic disease in which one or more of the following applies: (1) **relapsed or refractory (R/R) disease** after at least one line of disease-directed therapy; (2) the presence of **adverse-risk genetic or cytogenetic features** (for example, TP53 mutation or complex karyotype) that portend poor response to standard approaches; and/or (3) **treatment-ineligible status** – patients who are ineligible for standard curative-intent approaches (including intensive induction chemotherapy or myeloablative allogeneic transplant) because of advanced age, frailty, comorbidity burden, or organ dysfunction. This working definition intentionally blends biological aggressiveness and real-world clinical constraints to reflect the dual challenges of disease biology and host vulnerability that characterize many older adults with leukemia^[9]^.

## Acute myeloid leukemia

Late-stage AML in older adults is shaped by a convergence of adverse tumor biology and diminished host reserve. The older AML patient population disproportionately carries high-risk molecular lesions (TP53, ASXL1, and RUNX1) and often evolves from antecedent myelodysplastic syndromes or clonal hematopoiesis. These features are associated with primary chemoresistance, higher rates of early mortality, and short remission durations. Standard approaches must therefore be tailored. For patients fit for intensive therapy, anthracycline- and cytarabine-based induction remains an option but is frequently precluded by comorbidity and frailty. For the majority who are older and/or unfit, **low-intensity regimens** – notably hypomethylating agents (azacitidine or decitabine) combined with the BCL-2 inhibitor **venetoclax** – have emerged as preferred front-line strategies because they produce meaningful remissions with an acceptable toxicity profile. Select targeted agents (FLT3, IDH1/2 inhibitors) are indicated when actionable mutations are present and can be combined or sequenced with lower-intensity backbones. CPX-351 is an evidence-based option for therapy-related AML or AML with myelodysplasia-related changes in appropriately selected older patients^[3,10]^. Supportive care is integral and often determines outcomes: aggressive infection surveillance and prophylaxis during prolonged cytopenias, transfusion strategies that balance symptom control and alloimmunization risk, nutritional and rehabilitation interventions to counter deconditioning, and early integration of palliative care to manage symptoms and treatment decisions. Comprehensive geriatric assessment (CGA) should inform intensity selection (full-dose vs reduced-intensity vs best supportive care) because physiologic age often diverges from chronological age. In selected fit older adults, reduced-intensity allogeneic transplant remains a potential curative option but requires careful assessment of comorbidity, frailty, and psychosocial support^[11]^.

## Acute lymphoblastic leukemia

In older adults, acute lymphoblastic leukemia (ALL) frequently presents with biological high-risk features – most notably a greater prevalence of Philadelphia chromosome (BCR-ABL1) positivity – and poorer tolerance of intensive pediatric-style regimens. Age-associated marrow microenvironment changes and decreased immune competence contribute to more aggressive clinical courses and higher treatment-related morbidity^[12]^. Therapeutic paradigms for late-stage ALL in older patients emphasize **biology-directed and less myelotoxic approaches.** For Ph-positive disease, combining **tyrosine kinase inhibitors (TKIs)** with attenuated chemotherapy or steroid-based regimens achieves high response rates while reducing early toxicity. For Ph-negative disease or R/R settings, **immunotherapies** (blinatumomab and inotuzumab ozogamicin) enable chemotherapy-sparing strategies that can induce deep remissions in frail patients; these agents have specific toxicity profiles (neurotoxicity for blinatumomab; veno-occlusive disease risk with inotuzumab) that demand vigilant monitoring. Chimeric antigen receptor (CAR) T-cell therapies have produced remarkable responses in select R/R cases but pose logistical, toxicity (cytokine release syndrome, neurotoxicity), and durability challenges in older, comorbid patients^[13]^. Supportive care priorities include central nervous system (CNS) prophylaxis adapted to tolerance, prophylactic antimicrobials during periods of neutropenia, metabolic management (steroids and TKIs can worsen glycemic and cardiovascular status), and thrombosis risk mitigation, especially with antibody–drug conjugates. Again, CGA and close coordination with geriatrics and palliative care guide whether the goal is disease control with acceptable toxicity or symptom-directed care^[14]^.

## Chronic lymphocytic leukemia

CLL is predominantly a disease of older adults and exemplifies how effective targeted therapies have shifted the balance toward chronic disease management even in late-stage settings. Age-related biology in CLL includes a higher relative frequency of adverse prognostic markers such as TP53 disruption and unmutated IGHV, which predict inferior responses to chemoimmunotherapy and guide the use of targeted agents^[15]^. Current standard-of-care options favor **oral targeted therapies** – Bruton’s tyrosine kinase (BTK) inhibitors (ibrutinib, acalabrutinib, and zanubrutinib) and BCL-2 inhibitor **venetoclax** (often given with anti-CD20 antibody for fixed-duration therapy) – because they avoid the cytopenias and infectious risks of conventional chemotherapy. In late-stage CLL (R/R disease or patients with adverse genetics), continuous BTK inhibition or finite venetoclax-based regimens can achieve durable control; choice is individualized by mutation profile, comorbidities (especially cardiac disease and bleeding risk with BTK inhibitors), and polypharmacy (drug–drug interactions via Cytochrome P450 [CYP] pathways). Real-world tolerability notes higher rates of atrial fibrillation and hypertension with some BTK inhibitors and tumor lysis syndrome risk during venetoclax initiation, mandating stepwise dose ramp-up and appropriate prophylaxis^[16]^. Supportive care focuses on infection prevention (vaccination, immunoglobulin replacement for recurrent infections when indicated), fall and bleeding risk mitigation, and the management of cardiovascular comorbidities that may be exacerbated by therapy. Shared decision-making should weigh continuous therapy burdens against fixed-duration strategies that can permit treatment-free intervals important for quality of life in older adults^[13]^.

## Chronic myeloid leukemia

Chronic myeloid leukemia’s biology – driven by the BCR-ABL1 fusion – is generally consistent across ages, but older patients face greater comorbidity burdens and higher likelihood of treatment intolerance. In late-stage contexts (accelerated phase, blast crisis, or resistance to multiple TKIs), the disease behaves more aggressively and requires complex therapeutic sequencing^[17]^. First-line management in older adults still centers on **BCR-ABL1-targeted TKIs** (imatinib and later-generation agents), chosen based on side-effect profiles and individual comorbidities. In late-stage or TKI-resistant disease, newer TKIs or combination strategies may be employed, and hematopoietic stem cell transplant remains a consideration for selected patients who can tolerate the procedure. In blast crisis, therapy often mirrors acute leukemia approaches with consideration of targeted agents and urgent supportive measures^[18]^. Supportive care priorities include rigorous monitoring of molecular response (BCR-ABL1 transcript levels) to guide therapy changes, proactive management of TKI toxicities (cardiac, metabolic, and pleural effusions), and attention to adherence challenges in older adults with polypharmacy or cognitive impairment. For many older patients who achieve deep molecular responses, treatment discontinuation strategies are an area of active research and may offer quality-of-life benefits, but such approaches require reliable monitoring infrastructure^[19,20]^.

## Diagnostic challenges in late-stage leukemias among elderly patients

The global demographic shift toward an aging population has profound implications for the epidemiology and management of cancer. Leukemia, a heterogeneous group of hematological malignancies, disproportionately affects older adults, with the highest incidence and mortality rates observed in individuals aged 65 years and above. As life expectancy increases worldwide, the burden of late-stage leukemias among the elderly is expected to rise substantially, posing significant clinical and public health challenges. These challenges necessitate a rethinking of traditional oncology paradigms to better meet the complex needs of this vulnerable population^[9,10]^. Late-stage leukemias in aging populations are often characterized by aggressive disease biology, delayed diagnosis, and frequent comorbid conditions. Older adults tend to present with more advanced disease due to nonspecific symptoms that may be mistaken for normal aging or other chronic illnesses, leading to diagnostic delays. Additionally, physiological changes related to aging, such as diminished bone marrow reserve and immune senescence, contribute to poorer disease control and treatment resistance. These factors combine to complicate clinical management and worsen prognosis compared to younger patients^[3,11]^.

The treatment of late-stage leukemias in elderly patients is further complicated by the coexistence of frailty, multiple comorbidities, and altered pharmacokinetics, which affect the safety and efficacy of standard chemotherapy and hematopoietic stem cell transplantation. Conventional aggressive treatments often carry high risks of toxicity and treatment-related mortality, prompting clinicians to seek less intensive or alternative therapeutic strategies. However, the evidence base guiding treatment decisions in this group remains limited, as older adults are frequently underrepresented in clinical trials^[12,13]^. Personalized medicine offers promise in addressing the heterogeneity of leukemia in aging populations by tailoring therapies based on molecular and clinical characteristics, as well as patient preferences and functional status. CGA is an essential tool in this approach, enabling clinicians to evaluate frailty, cognitive function, nutrition, and psychosocial factors to optimize treatment selection and supportive care. When combined with advances in molecular diagnostics and targeted therapies, personalized medicine can improve treatment tolerance and outcomes for elderly leukemia patients^[14]^.

Integrated care models that bridge oncology, geriatrics, palliative care, and psychosocial support are critical to delivering holistic care to aging patients with late-stage leukemias. Such models promote multidisciplinary collaboration, ensuring that medical treatment is balanced with quality-of-life considerations and symptom management. Coordinated care pathways can also enhance communication between providers, patients, and families, facilitating shared decision-making and adherence to therapy^[6]^. From a public health perspective, the increasing prevalence of late-stage leukemias in older adults demands systemic responses that improve early diagnosis, access to novel therapies, and supportive care infrastructure. Health systems in many regions face resource constraints, workforce shortages, and inequities that disproportionately affect elderly patients, particularly in underserved areas. Addressing these barriers through policy reforms, investment in health technologies, and workforce development is essential to ensuring equitable and effective leukemia care^[15]^.

## Therapeutic complexities and personalized medicine

Treating late-stage leukemias in aging populations presents a unique set of therapeutic challenges that stem from both disease- and patient-specific factors. Elderly patients frequently exhibit reduced physiological reserve, frailty, and multiple comorbidities such as cardiovascular disease, diabetes, and renal impairment, which collectively increase the risk of treatment-related toxicity and adverse outcomes. Moreover, aging alters pharmacokinetics and pharmacodynamics, leading to variable drug absorption, metabolism, and clearance. These factors necessitate a careful balancing between treatment efficacy and tolerability, often precluding the use of standard intensive chemotherapy regimens commonly employed in younger patients^[13,16]^. The heterogeneity of leukemia biology in older adults further complicates treatment selection. Molecular and cytogenetic profiles of leukemic cells often differ with age, with elderly patients more likely to harbor adverse-risk mutations and chromosomal abnormalities that confer resistance to conventional therapies. These biological differences underscore the need for personalized medicine approaches that integrate molecular diagnostics to guide targeted therapies and prognostication. Advances in next-generation sequencing (NGS) and other genomic technologies have facilitated the identification of actionable mutations, enabling tailored treatment strategies that may improve outcomes while reducing toxicity^[17,18]^.

CGA is a cornerstone of personalized care for elderly leukemia patients, offering a multidimensional evaluation of physical function, cognition, comorbidity burden, psychological health, and social support. Incorporating CGA into clinical decision-making helps stratify patients based on fitness and vulnerability, guiding the intensity of treatment and supportive care interventions. For example, fit elderly patients may tolerate moderately intensive therapies or clinical trial enrollment, whereas frail individuals might benefit more from low-intensity regimens or best supportive care. CGA also aids in identifying modifiable factors such as nutritional deficits and polypharmacy, which can be optimized to enhance treatment tolerance^[19]^. The emergence of targeted therapies and immunotherapies has expanded therapeutic options for elderly patients with late-stage leukemia, offering the potential for improved efficacy with fewer side effects compared to traditional chemotherapy. Agents such as hypomethylating agents, FLT3 inhibitors, IDH inhibitors, and BCL-2 antagonists have demonstrated activity in older populations, often with more manageable toxicity profiles. However, access to these novel treatments remains uneven, particularly in resource-limited settings, highlighting the need for equitable distribution and cost-containment strategies^[20]^.

## Integrated care models: bridging oncology, geriatrics, and palliative care

The management of late-stage leukemias in aging populations demands a shift from traditional, disease-centered approaches to integrated care models that holistically address the complex medical, functional, and psychosocial needs of elderly patients. Aging patients often experience a convergence of cancer-related symptoms, treatment side effects, comorbidities, and geriatric syndromes such as frailty and cognitive impairment, which necessitates coordinated care across multiple specialties. Integrated care models that seamlessly bridge oncology, geriatrics, and palliative care are increasingly recognized as essential to improving outcomes and quality of life^[21]^. Oncology and geriatrics collaboration is central to effective integrated care for elderly leukemia patients. Geriatricians bring expertise in evaluating functional status, comorbidities, polypharmacy, and vulnerability to treatment toxicity, complementing oncologists’ focus on disease biology and therapy. Early involvement of geriatric assessment enables risk stratification and informs individualized treatment plans that balance efficacy with safety. Multidisciplinary tumor boards, including geriatricians, oncologists, pharmacists, and social workers, facilitate shared decision-making, ensuring that treatment goals align with patient preferences and overall health status^[22]^.

Palliative care integration throughout the disease trajectory is another critical component of comprehensive care. Palliative care teams address symptom management, psychological support, and advance care planning, improving patient comfort and reducing hospitalizations. In late-stage leukemias, symptoms such as pain, fatigue, infections, and bleeding complications are common and often challenging to manage. Timely palliative interventions can mitigate suffering and enhance quality of life, even alongside disease-modifying therapies. Importantly, early palliative care involvement has been shown to improve both symptom burden and survival in various cancers, underscoring its value in leukemia care^[23]^. Integrated care models also emphasize coordination and communication among health care providers, patients, and caregivers. This includes standardized care pathways, shared electronic health records, and dedicated care coordinators or nurse navigators who facilitate appointments, monitor treatment adherence, and provide education. Such systems reduce fragmentation, prevent duplication of services, and improve patient satisfaction. Supportive care services addressing nutrition, physical rehabilitation, mental health, and social determinants of health are often integrated to comprehensively support patients and families^[24]^. Beyond clinical care, integrated models foster a patient-centered approach that respects individual goals, cultural values, and quality-of-life priorities. Elderly patients with late-stage leukemia frequently face complex decisions about balancing life-prolonging treatments with comfort and dignity. Multidisciplinary teams trained in communication skills and ethics can support these decisions through shared decision-making frameworks, advance directives, and goals-of-care discussions (Table 1 and Table 2)^[25]^.

**Table 1:** Integrated care models: bridging oncology, geriatrics, and palliative care

Component	Description	Key stakeholders	Expected outcomes
Comprehensive geriatric assessment	Multidimensional tool assessing functional status, cognition, nutrition, polypharmacy, and support systems	Geriatricians, oncologists, and nurses	Individualized treatment plans, early frailty detection, and reduced treatment toxicity
Personalized oncology protocols	Tailoring chemotherapy, targeted therapy, or supportive care based on biological and functional age	Hematologists and molecular pathologists	Improved survival, better treatment tolerance, and minimized adverse effects
Palliative and supportive care integration	Early incorporation of symptom control, psychosocial support, and goals-of-care discussions	Palliative care teams, social workers, and caregivers	Enhanced quality of life, reduced hospital admissions, and increased patient satisfaction
Multidisciplinary care teams	Coordinated care involving oncology, geriatrics, nursing, pharmacy, nutrition, and social services	Multidisciplinary team and hospital administration	Holistic decision-making, continuity of care, and shared accountability
Home-based and telehealth services	Decentralized care delivery models including virtual monitoring, mobile labs, and home chemotherapy	Primary care providers, telemedicine units, and outreach teams	Increased access, reduced travel burden and timely interventions
Decision support tools and technology	Use of AI, electronic health records, and predictive analytics to guide risk stratification	Data scientists, clinicians, and IT specialists	Evidence-based decisions, proactive care adjustments, and personalized risk prediction
Community and family engagement	Inclusion of family and community networks in care coordination and education	Caregivers and community health workers	Improved adherence, culturally sensitive care, and enhanced emotional support

**Table 2:** Comprehensive geriatric assessment domains and commonly used tools for older adults with late-stage leukemia

Domain	Purpose/focus	Common tools/scales	Clinical utility in leukemia care
Functional status	Evaluates ability to perform activities of daily living (ADLs) and instrumental ADLs (IADLs).	Katz ADL Scale and Lawton IADL Scale	Identifies need for supportive services, predicts treatment tolerance, and guides therapy intensity.
Comorbidity burden	Assesses chronic illnesses that may impact prognosis or therapy risk.	Hematopoietic Cell Transplantation–Comorbidity Index and Charlson Comorbidity Index	Stratifies transplant eligibility, estimates non-relapse mortality, and informs regimen selection.
Frailty/vulnerability	Screens for global physiologic reserve and vulnerability to stressors.	G8 Screening Tool, Clinical Frailty Scale, and Fried Frailty Criteria	Rapidly detects frailty to trigger full CGA and supportive interventions.
Cognition	Detects cognitive impairment affecting decision-making and adherence.	Mini-Mental State Examination and Montreal Cognitive Assessment	Guides consent processes, medication management, and caregiver involvement.
Psychological health	Screens for depression, anxiety, and emotional distress.	Geriatric Depression Scale and Hospital Anxiety and Depression Scale	Identifies need for psychosocial support and early palliative care integration.
Nutrition	Evaluates nutritional risk, weight loss, and sarcopenia.	Mini Nutritional Assessment and Malnutrition Universal Screening Tool	Predicts chemotherapy toxicity and infection risk; informs dietary interventions.
Polypharmacy/medication review	Identifies inappropriate or interacting medications.	Beers Criteria and STOPP/START	Reduces adverse drug interactions, particularly with targeted therapies and antifungals.
Social support/environment	Assesses caregiver availability, social isolation, and economic barriers.	Social Support Questionnaire and caregiver burden scales	Guides discharge planning, home transfusion programs, and community resource referral.

## Public health implications and strategies

The rising incidence of late-stage leukemias in aging populations represents a significant public health challenge that extends beyond clinical management to encompass prevention, early detection, health equity, and resource allocation. As the global population ages, health systems worldwide must prepare to address the increasing burden of hematological malignancies while ensuring that elderly patients receive timely and appropriate care. This requires coordinated public health strategies that integrate clinical innovation with health policy, infrastructure development, and community engagement^[26,27]^. One of the key public health concerns is the underdiagnosis and delayed presentation of leukemia in older adults. Nonspecific symptoms and comorbidities often mask disease onset, leading to late-stage diagnosis when therapeutic options are limited and prognosis poor. Public health initiatives should focus on raising awareness among health care providers and the public about leukemia symptoms in the elderly, promoting routine hematological screening in high-risk groups, and improving access to diagnostic services. Early detection programs can facilitate timely intervention and improve survival rates^[28]^.

Health disparities and inequities in access to diagnostics, novel therapies, and specialized care are prevalent among elderly leukemia patients, particularly in low-resource and underserved regions. Socioeconomic factors, geographic barriers, and limited health infrastructure contribute to these disparities, exacerbating morbidity and mortality. Public health strategies must prioritize equitable resource distribution, capacity building, and health system strengthening to ensure that elderly patients, regardless of location or socioeconomic status, benefit from advances in leukemia care. Partnerships with non-governmental organizations, community health workers, and telemedicine platforms can extend reach and support^[29]^. Workforce training and interdisciplinary collaboration are essential components of an effective public health response. Expanding education and training in geriatric oncology and palliative care for health care professionals will improve competency in managing the complex needs of elderly leukemia patients. Establishing multidisciplinary teams and referral networks enhances care coordination and patient outcomes. Additionally, fostering research focused on aging populations will generate evidence to inform guidelines and policy^[30]^.

Policy interventions that support integrated care models and personalized medicine approaches are critical for sustainable impact. These include reimbursement frameworks that incentivize CGAs coverage for targeted therapies and investment in supportive care services. Health information systems should be optimized to facilitate data collection, monitoring, and evaluation of leukemia care programs, enabling continuous quality improvement and policy refinement^[30]^. Community engagement and patient empowerment are also vital public health strategies. Educating patients and caregivers about disease management, treatment options, and supportive care promotes adherence and shared decision-making. Culturally sensitive interventions that respect diverse values and preferences enhance acceptability and effectiveness. Furthermore, addressing social determinants of health such as nutrition, housing, and social support can mitigate vulnerabilities that worsen leukemia outcomes in the elderly^[31,32]^.

## Innovations

Recent advances in biomedical research and technology have ushered in a new era of innovation aimed at overcoming the challenges of managing late-stage leukemias in aging populations. These innovations span molecular diagnostics, targeted therapeutics, digital health, and care delivery models, all contributing to more precise, effective, and patient-centered care for elderly leukemia patients^[15]^. Molecular and genomic technologies have revolutionized leukemia diagnosis and treatment personalization. NGS and other high-throughput platforms enable detailed characterization of leukemia subtypes at the genetic and epigenetic levels. This allows identification of actionable mutations and prognostic biomarkers that guide targeted therapy selection, risk stratification, and monitoring of minimal residual disease. Such precision medicine approaches are especially valuable in elderly patients, as they enable tailoring treatment intensity to disease biology and individual tolerance, minimizing unnecessary toxicity^[33]^.

Targeted therapies and immunotherapies represent a transformative leap in leukemia treatment for older adults. Agents such as FLT3 inhibitors, IDH1/2 inhibitors, BCL-2 inhibitors (e.g., venetoclax), and monoclonal antibodies have shown efficacy with improved safety profiles compared to conventional chemotherapy. Additionally, cellular therapies like CAR T-cell therapy and bispecific T-cell engagers are expanding therapeutic horizons, although their use in elderly populations requires further study to optimize safety and feasibility. These novel treatments offer hope for better outcomes and reduced treatment-related morbidity^[34]^. Digital health and telemedicine innovations have increasingly facilitated access to specialized leukemia care, particularly for elderly patients in remote or underserved areas. Teleconsultations, remote monitoring of symptoms and blood counts, and electronic health records improve continuity of care, patient engagement, and early identification of complications. Wearable devices and mobile health applications can track patient-reported outcomes and functional status, aiding personalized treatment adjustments and supportive care^[35,36]^.

**Figure 1.**
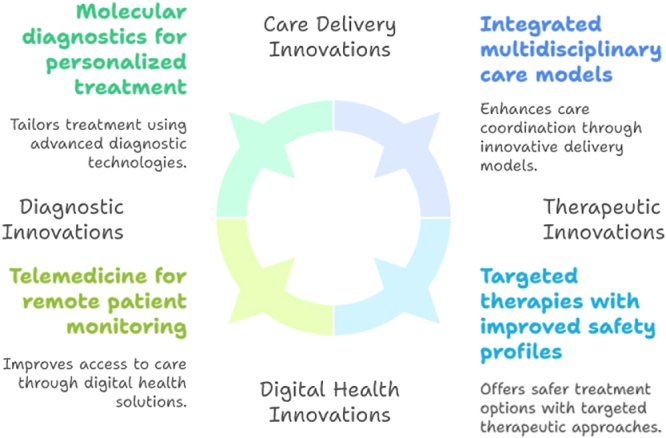
Innovations in leukemia management for aging populations.

**Figure 2.**
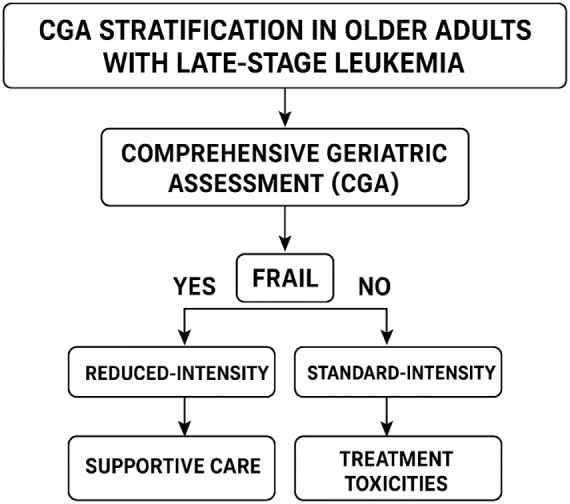
A simplified algorithm showing how comprehensive geriatric assessment (CGA) stratification informs treatment intensity and supportive-care triggers.

In the realm of care delivery, integrated multidisciplinary models leveraging innovations in communication and coordination are emerging as best practices. Care pathways supported by clinical decision support systems and data analytics enable real-time risk assessment and treatment optimization. Moreover, advances in supportive care – including novel antiemetics, infection prophylaxis, and nutritional interventions – enhance treatment tolerability and quality of life for elderly patients undergoing leukemia therapy^[37]^. Research innovations are also addressing gaps in clinical trial inclusion of elderly patients. Adaptive trial designs, real-world evidence generation, and patient-centered outcome measures are increasingly incorporated to better reflect the heterogeneity of aging populations. These efforts aim to generate robust data that can inform age-appropriate treatment guidelines and reduce uncertainty in clinical decision-making (Fig. 1 and Fig. 2)^[38–40]^.

## Linking integrated care models to measurable outcomes

Integrated care models have become increasingly relevant in the management of late-stage leukemias among older adults, largely because fragmented care often leads to preventable complications, reduced treatment adherence, and poorer survival. When oncology, geriatrics, primary care, palliative services, and psychosocial support are coordinated, patients benefit from a more stable and predictable care pathway. This coordinated structure enables the systematic monitoring of symptoms, functional status, comorbidities, and treatment responses, all of which are essential for optimizing outcomes in vulnerable aging populations^[41]^. Evidence from geriatric oncology programs shows that integrating CGA into treatment planning can reduce treatment-related toxicity, lower hospitalization rates, and improve tolerance of leukemia-directed therapy. Similarly, interdisciplinary case conferences have been associated with clearer decision-making, better alignment of treatment intensity with patient goals, and earlier recognition of complications that require intervention. These improvements translate into measurable benefits such as enhanced quality of life, fewer emergency admissions, and increased likelihood of completing recommended therapy^[42]^.

Integrated models also strengthen communication across health care teams, which support more accurate prognostication and reduce the risk of duplicative investigations or conflicting treatment plans. In advanced leukemia, where symptom burden is often high, early involvement of palliative care improves management of pain, fatigue, and emotional distress. Studies have shown that early palliative input can increase patient-reported satisfaction, reduce caregiver strain, and, in some cases, extend survival by minimizing aggressive but non-beneficial interventions at the end of life^[43]^. From a systems perspective, integrated care frameworks facilitate the collection of standardized data on treatment outcomes, functional decline, and health service utilization. These metrics help health care institutions evaluate program effectiveness and refine service delivery. Overall, the link between integrated care and measurable improvements in clinical, functional, and patient-reported outcomes underscores the importance of adopting coordinated, patient-centered models for elderly individuals facing late-stage leukemia^[44,45]^.

## Conclusion

Late-stage leukemias in aging populations present a uniquely complex intersection of biological vulnerability, cumulative comorbidities, delayed diagnosis, and reduced treatment tolerance. As global demographics shift toward an older population, these challenges will only intensify, underscoring the need for a reorientation of clinical practice and public health strategy. Although advances in molecular profiling and targeted therapies have expanded treatment possibilities, meaningful progress requires more than therapeutic innovation. Integrated, patient-centered models of care that combine oncological expertise with geriatric assessment, coordinated supportive services, and early palliative involvement are essential for improving outcomes and preserving quality of life. Strengthening diagnostic capacity, standardizing care pathways, and ensuring equitable access to emerging treatments remain pressing public health priorities. By aligning personalized medicine with coordinated multidisciplinary care, health care systems can better meet the needs of older adults facing late-stage leukemia and advance toward more compassionate, effective, and sustainable models of cancer care.

## References

[1] Gu YF Lin FP Epstein RJ. How aging of the global population is changing oncology. Ecancermedicalscience 2021;15:ed119. 35211208 10.3332/ecancer.2021.ed119 PMC8816510

[2] Li L Shan T Zhang D. Nowcasting and forecasting global aging and cancer burden: analysis of data from the GLOBOCAN and Global Burden of Disease Study. J Natl Cancer Cent 2024;4:223–32. 39281725 10.1016/j.jncc.2024.05.002 PMC11401500

[3] Almeida AM Ramos F. Acute myeloid leukemia in the older adults. Leuk Res Rep 2016;6:1–7. 27408788 10.1016/j.lrr.2016.06.001 PMC4927655

[4] Stone RM Lindsley C. Older adults with acute myeloid leukemia treated with intensive chemotherapy: “old” prognostic algorithms may not apply. Haematologica 2018;103:1758–59. 30381416 10.3324/haematol.2018.201848 PMC6278980

[5] Appelbaum FR. What is the impact of hematopoietic cell transplantation (HCT) for older adults with acute myeloid leukemia (AML)? best pract. Res Clin Haematol 2008;21:667–75. 10.1016/j.beha.2008.06.005 PMC3163849 19041606

[6] Nipp RD Subbiah IM Loscalzo M. Convergence of geriatrics and palliative care to deliver personalized supportive care for older adults with cancer. J Clin Oncol 2021;39:2185–94. 34043435 10.1200/JCO.21.00158 PMC8260927

[7] Bradley N McConnell T Blair C. Integrated palliative care and oncology: a realist synthesis. BMC Med 2025;23:272. 40346564 10.1186/s12916-025-04083-1 PMC12065255

[8] Albarqi MN. Assessing the impact of multidisciplinary collaboration on quality of life in older patients receiving primary care: cross sectional study. Healthcare 2024;12:1258. 38998793 10.3390/healthcare12131258 PMC11240966

[9] Krok-Schoen JL Fisher JL Stephens JA. Incidence and survival of hematological cancers among adults ages ≥75 years. Cancer Med 2018;7:3425–33. 29654631 10.1002/cam4.1461 PMC6051144

[10] Huang J Chan SC Ngai CH. Disease burden, risk factors, and trends of leukaemia: a global analysis. Front Oncol 2022;12:904292. 35936709 10.3389/fonc.2022.904292 PMC9355717

[11] Storey S Gray TF Bryant AL. Comorbidity, physical function, and quality of life in older adults with acute myeloid leukemia. Curr Geriatr Rep 2017;6:247–54. 29479516 10.1007/s13670-017-0227-8 PMC5820770

[12] Krug U Büchner T Berdel WE. The treatment of elderly patients with acute myeloid leukemia. Dtsch Arztebl Int 2011;108:863–70. 22259641 10.3238/arztebl.2011.0863 PMC3258577

[13] Eleni LD Nicholas ZC Alexandros S. Challenges in treating older patients with acute myeloid leukemia. J Oncol 2010;2010:943823. 20628485 10.1155/2010/943823 PMC2902223

[14] O’Donovan A Leech M. Personalised treatment for older adults with cancer: the role of frailty assessment. Tech Innov Patient Support Radiat Oncol 2020;16:30–38. 33102819 10.1016/j.tipsro.2020.09.001 PMC7568178

[15] Obeagu EI. Advancing leukemia diagnosis and treatment: WHO-supported laboratory innovations in Africa- A narrative review. Blood Lymphat Cancer 2025;15:47–67. 40584840 10.2147/BLCTT.S518005 PMC12205951

[16] Sawalha Y Advani AS. Management of older adults with acute lymphoblastic leukemia: challenges & current approaches. Int J Hematol Oncol 2018;7:IJH02. 30302234 10.2217/ijh-2017-0023 PMC6176956

[17] DiNardo CD Cortes JE. Mutations in AML: prognostic and therapeutic implications. Hematology Am Soc Hematol Educ Program 2016;2016:348–55. 27913501 10.1182/asheducation-2016.1.348 PMC6142505

[18] Prassek R-T VV Sauerland M Herold MC. Genetics of acute myeloid leukemia in the elderly: mutation spectrum and clinical impact in intensively treated patients aged 75 years or older. Haematologica 2018;103:1853–61. 29903761 10.3324/haematol.2018.191536 PMC6278991

[19] Overcash J Ford N Kress E. Comprehensive geriatric assessment as a versatile tool to enhance the care of the older person diagnosed with cancer. Geriatrics (Basel) 2019;4:39. 31238518 10.3390/geriatrics4020039 PMC6630523

[20] Kayser S Levis MJ. Advances in targeted therapy for acute myeloid leukaemia. Br J Haematol 2018;180:484–500. 29193012 10.1111/bjh.15032 PMC5801209

[21] Sutton LM Demark-Wahnefried W Clipp EC. Management of terminal cancer in elderly patients. Lancet Oncol 2003;4:149–57. 12623360 10.1016/s1470-2045(03)01019-2

[22] Karnakis T Souza PMR Kanaji AL. The role of geriatric oncology in the care of older people with cancer: some evidence from Brazil and the world. Rev Assoc Med Bras (1992) 2024;70:e2024S118. 38865538 10.1590/1806-9282.2024S118 PMC11164271

[23] Hui D Bruera E. Integrating palliative care into the trajectory of cancer care. Nat Rev Clin Oncol 2016;13:159–71. 26598947 10.1038/nrclinonc.2015.201 PMC4772864

[24] Foo C Yan JY Chan ASL. Identifying key themes of care coordination for patients with chronic conditions in singapore: a scoping review. Healthcare (Basel) 2023;11:1546. 37297686 10.3390/healthcare11111546 PMC10252872

[25] Oliva EN, and Almeida A. Determining treatment pathways for older patients with acute myeloid leukemia: patient and clinician perspectives. Expert Rev Hematol 2025;18:595–604. 10.1080/17474086.2025.2521397 40525509

[26] Hu C Chen W Zhang P. Global, regional, and national burden of leukemia: epidemiological trends analysis from 1990 to 2021. PLoS One 2025;20:e0325937. 40569983 10.1371/journal.pone.0325937 PMC12200851

[27] Han X Yun Z Liu Z. Global, regional, and national burden of acute leukemia and its risk factors from 1990 to 2021 and predictions to 2040: findings from the global burden of disease study 2021. Biomed Eng Online 2025;24:72. 40495176 10.1186/s12938-025-01403-7 PMC12150532

[28] Black GB Boswell L Harris J. What causes delays in diagnosing blood cancers? A rapid review of the evidence. Prim Health Care Res Dev 2023;24:e26. 37039465 10.1017/S1463423623000129 PMC10156470

[29] Nair N Schlumbrecht M. Existing health inequities in the treatment of advanced and metastatic cancers. Curr Oncol Rep 2024;26:1553–62. 39495424 10.1007/s11912-024-01617-3 PMC11646267

[30] Alhaddar M Falna H Sultan H. Towards enhancing palliative care competencies through comprehensive training for nurses and physicians in resource-limited settings: a cross-sectional study. BMC Nurs 2025;24:688. 40598507 10.1186/s12912-025-03412-2 PMC12210702

[31] Montori VM Ruissen MM Hargraves IG. Shared decision-making as a method of care. BMJ Evid Based Med 2023;28:213–17. 10.1136/bmjebm-2022-112068 PMC10423463 36460328

[32] Cipta DA Andoko D Theja A. Culturally sensitive patient-centered healthcare: a focus on health behavior modification in low and middle-income nations-insights from Indonesia. Front Med (Lausanne) 2024;11:1353037. 38681051 10.3389/fmed.2024.1353037 PMC11047771

[33] Pratiwi L Mashudi FH Ningtyas MC. Genetic profiling of acute and chronic leukemia via next-generation sequencing: current insights and future perspectives. Hematol Rep 2025;17:18. 40277842 10.3390/hematolrep17020018 PMC12026831

[34] Totiger TM Ghoshal A Zabroski J. Targeted therapy development in acute myeloid leukemia. Biomedicines 2023;11:641. 36831175 10.3390/biomedicines11020641 PMC9953553

[35] Ezeamii VC Okobi OE Wambai-Sani H. Revolutionizing healthcare: how telemedicine is improving patient outcomes and expanding access to care. Cureus 2024;16:e63881. 39099901 10.7759/cureus.63881 PMC11298029

[36] Shaffer KM Turner KL Siwik C. Digital health and telehealth in cancer care: a scoping review of reviews. Lancet Digit Health 2023;5:e316–e327. 37100545 10.1016/S2589-7500(23)00049-3 PMC10124999

[37] Jerjes W Harding D. Enhancing primary care through integrated care pathways: a convergence of theory and practice. Front Health Serv 2024;4:1432901. 39726896 10.3389/frhs.2024.1432901 PMC11669714

[38] Marzetti E Calvani R Coelho-Junior HJ. Advancing the methodology of clinical trials for aging populations: a call to innovation, inclusion, and global relevance. J Nutr Health Aging 2025;29:100587. 40412138 10.1016/j.jnha.2025.100587 PMC12172968

[39] Agha RA Mathew G Rashid R, and TITAN Group. Transparency in the reporting of Artificial Intelligence – the TITAN Guideline. Prem J Sci 2025;10:100082.

[40] Gangat N Dinardo CD. Newly diagnosed acute myeloid leukemia in unfit patients: 2026 treatment algorithms. Blood Cancer J 2025;15:139. 40818987 10.1038/s41408-025-01346-1 PMC12357898

[41] Robbins-Welty GA Webb JA Shalev D. Advancing palliative care integration in hematology: building upon existing evidence. Curr Treat Options Oncol 2023;24:542–64. 37017909 10.1007/s11864-023-01084-1 PMC10074347

[42] Tarchand GR Morrison V Klein MA. Use of comprehensive geriatric assessment in oncology patients to guide treatment decisions and predict chemotherapy toxicity. Fed Pract 2021;38:S22–S28. 34177238 10.12788/fp.0128 PMC8223735

[43] Mäkitie A Saarto T. Integration of palliative care as a part of the multidisciplinary management of patients with head and neck cancer. Oncol Ther 2025;13:541–46. 40699445 10.1007/s40487-025-00363-1 PMC12378248

[44] Bautista MA Nurjono M Lim YW. Instruments measuring integrated care: a systematic review of measurement properties. Milbank Q 2016;94:862–917. 27995711 10.1111/1468-0009.12233 PMC5192798

[45] Isaacs AN Mitchell EKL. Mental health integrated care models in primary care and factors that contribute to their effective implementation: a scoping review. Int J Ment Health Syst 2024;18:5. 38331913 10.1186/s13033-024-00625-x PMC10854062

